# Tribological and Corrosion Properties of Coatings of Ultradisperse TiB_2_-TiAl Electrodes with Nanosized Additives Deposited on Ti-Gr2 by Non-Contact Electrospark Deposition

**DOI:** 10.3390/ma19081652

**Published:** 2026-04-21

**Authors:** Georgi Kostadinov, Antonio Nikolov, Yavor Sofronov, Todor Penyashki, Valentin Mishev, Boriana Tzaneva, Rayna Dimitrova, Krum Petrov, Radoslav Miltchev, Todor Gavrilov

**Affiliations:** 1 Faculty of Industrial Technology, Technical University of Sofia, 1756 Sofia, Bulgaria; tpeniashki@abv.bg; 2 Department of Material Science and Technology of Materials, Faculty of Industrial Technology, Technical University of Sofia, 1756 Sofia, Bulgaria; anikolov@tu-sofia.bg (A.N.); v_mishev@tu-sofia.bg (V.M.); r_dimitrova@tu-sofia.bg (R.D.); kpetrov@tu-sofia.bg (K.P.); 3 Department of Theory of Mechanisms and Machines, Faculty of Industrial Technology, Technical University of Sofia, 1756 Sofia, Bulgaria; rmiltchev@tu-sofia.bg; 4 Center of Excellence “Mechatronics and Clean Technology”—Campus Studentski Grad, Technical University of Sofia, 1756 Sofia, Bulgaria; borianatz@tu-sofia.bg; 5 Department of Chemistry, Faculty of Electronic Engineering and Technologies, Technical University of Sofia, 1756 Sofia, Bulgaria; 6 Department of Manufacturing Technology and Systems, Faculty of Industrial Technology, Technical University of Sofia, 1756 Sofia, Bulgaria; tgavrilov@tu-sofia.bg

**Keywords:** local electrospark deposition, titanium, wear resistance, corrosion resistance, corrosion characteristics, micropores

## Abstract

In this work, the tribological and corrosion behavior of commercially pure titanium—Ti-Gr2 with coatings obtained by mechanized contactless local electrospark deposition (LESD) with low pulse energy and a rotating electrode of TiB_2_-TiAl reinforced with ZrO_2_ and NbC nanoparticles was investigated. The current research is driven by the need for improved corrosion and abrasion resistance of titanium surfaces in automotive components, shipbuilding, aerospace, petrochemical and many other industrial and domestic areas. This work is a continuation of our previous study, in which the dependences of the relief, roughness, thickness, microhardness, composition and structure of the coatings obtained with this electrode on the electrical parameters of the LESD mode were studied and analyzed. In this work, the influence of the pulse parameters of the LESD process (respectively, roughness, thickness, composition and structure of the coatings) on the tribological and corrosion characteristics of the coatings has been investigated and the possibility of simultaneous protection of titanium surfaces from wear and corrosion has been demonstrated. Coatings containing nanocrystalline and amorphous-like structures have been formed, with synthesized new compounds and phases, and with increased hardness up to 13 GPa, low roughness Ra = 1.5–3 μm, thickness 8–20 μm and minimal structural defects. By comparing the potentiodynamic polarization curves, polarization resistance, electrochemical impedance and tribological characteristics of the coated surfaces, it has been established that their corrosion resistance increases by more than 1–2 orders of magnitude and their wear resistance during friction increases by 4–5 times compared to those of the substrate. Appropriate values of the electrical parameters of the LESD mode are presented, which allow obtaining uniform coatings with reduced roughness and structural defects, with predictable thickness, roughness and hardness, and with maximized corrosion and abrasive wear resistance to allow for uniform coatings with reduced roughness and structural defects, with predictable thickness, roughness and hardness, and with maximized corrosion and abrasive wear resistance.

## 1. Introduction

Titanium and its alloys are desirable materials for many industrial applications, but their low hardness and wear resistance limit their use. One of the main approaches to improve their low hardness and wear resistance and to respond to the progressively growing interests in their use in various industrial sectors is the widespread use of protective and wear-resistant coatings. However, for the most part, these methods are not always effective and suitable for titanium products, since most of them require complex and expensive technologies and equipment and high costs. They also do not always meet a number of requirements for adhesion, hardness, thickness, roughness, environmental pollution, dimensions and geometry of the processed products, etc. Among these methods, electrospark deposition (ESD) stands out both for its extremely low cost, environmental friendliness; versatility; simple, cheap, compact and portable equipment; easy and flexible technologies; and high efficiency at extremely low costs [1,2,3], as well as for the possibilities of successfully overcoming the majority of the limitations that are characteristic of most of the other methods. ESD can also successfully overcome low surface hardness, low wear resistance, and limited oxidation resistance at high temperature [3].

This method allows the application of coatings from any conductive materials with high bonding strength to titanium substrates, the absence of thermal effects and deformation of the titanium substrate, the possibility of local processing of any conductive surface and ultra-fast local heating and cooling [3,4,5]. By changing the energy parameters of the ESD regimes, the characteristics and properties of the coatings can be changed in a wide range and coatings with highly effective physical, mechanical and tribological properties can be obtained, as shown in works [5,6,7] and the work of many other researchers. The high temperatures generated by the occurrence of short-term spark discharges of the order of microseconds, and the subsequent transfer of molten electrode material and its mixing with the locally molten material on the substrate surface, generate the formation of coatings firmly bonded to the substrate with new phases and compounds. The ultra-rapid cooling of the molten anode–cathode mixture in the time between two consecutive discharges leads to the obtaining of metastable phases and fine structures with inclusions of high-hard elements and compounds; and when using certain compositions and electrical process parameters, the formation of amorphous phases is also possible, which is reported in works [4,8,9,10]. The ultra-dispersed structure of ESD coatings of crystalline high-hardness particles embedded in a metal glassy matrix is attractive both in terms of abrasive wear resistance and in terms of corrosion and tribocorrosion protection, which is determining growing interest in the applications of the ESD method for strengthening the titanium and its alloys, as well as the progressive increase in the number of studies in this direction. However, most of those studies are focused on increasing the hardness and tribological characteristics of the coated surfaces, while improving corrosion resistance is the subject of relatively fewer studies [3,6]. ESD is most commonly used to strengthen and improve the wear resistance of steel surfaces by using hard alloy electrodes based on WC, TiC, TiCN, TiB_2_, Cr_2_C_3_, etc. [1,2,11,12].

It is known that the corrosion resistance of titanium and its alloys is mainly due to the oxide films formed on titanium surfaces [3,5,6,11]. However, the oxide layers are fragile and usually easily destroyed in the presence of mechanical loads, friction and elevated temperatures, which leads to rapid abrasive wear and corrosion of titanium surfaces. To improve the corrosion resistance of titanium and titanium alloys by ESD, researchers mainly use metals and metal alloys. Cadney et al. [8], Milligan et al. [9], Burkov et al. [10], use aluminum and aluminum alloys, with which they obtain coatings with amorphous-nanocrystalline structures, with improved corrosion resistance and wear resistance. Ti-Al alloys have been used to improve the corrosion resistance of titanium and its alloys and by other coating methods [13,14], and Kornienko et al. [15] reported on improving the corrosion resistance of titanium in ESD with a palladium electrode. The coatings of metals and metal alloys obtained by these and other authors, however, have lower hardness. (Burkov et al. [10], reported that the microhardness of TiAl coatings on Ti6Al4V alloy is 6.4–9.4 GPa) and the wear resistance of these coatings is lower than that when using hard alloy electrodes [16,17,18,19]. The corrosion resistance of hard alloy coatings, however, is usually lower than that of coatings of metals and metal alloys due to the presence of increased roughness and structural defects characteristic of hard alloy electrodes. In ESD of titanium surfaces with hard alloy electrodes, the coatings usually have higher roughness than that of the substrate, asperities and defects such as pores, microcracks and cavities, which reduce the effect of their abrasive and corrosion resistance and limit the practical application of the technology in many industrial and domestic areas, where simultaneous protection against corrosion and frictional wear, or tribocorrosion, is required. The increased hardness of hard alloy coatings can partially compensate for their lower corrosion resistance, but the difficulty in obtaining smooth and continuous coatings limits their use of this method to improve the corrosion resistance of titanium products.

Although the protection of titanium from corrosion has been the subject of scientific research, to date, no unified concept has been proposed in the literature on the influence of coating materials, coating characteristics, and energy parameters of ESD regimes on the corrosion mechanism and corrosion characteristics of coated surfaces. It is also not fully understood to what extent coatings with improved corrosion characteristics are able to improve the hardness and wear resistance of coated surfaces. Studies of the quality indicators of the surface layers (roughness, uniformity, continuity, microhardness, etc.) made it possible to establish that in order to achieve optimal simultaneous resistance to wear and corrosion, it is necessary to combine contradictory requirements such as hardness, strength and toughness, chemical and thermal resistance, and establish control of the characteristics and properties of the coatings and the correlations between the process parameters, composition, structure, properties and durability of the coated surfaces. The compositions of the coatings must combine sufficient thickness (>10 µm) [3,15,19,20], strong adhesion [3,6], high hardness, low roughness, a small number of structural defects, and high corrosion resistance.

Despite the availability of a research data, the potential of ESD for corrosion-resistant coatings on metals and metal alloys with low surface defects is still not well studied, and the growing need for improved hardness, wear resistance and corrosion resistance of titanium surfaces in many industries determines the need for research to create coatings with high corrosion and abrasion resistance. Therefore, there is a need to continue and expand research aimed at using the easy, environmentally friendly and accessible ESD method to create coatings with reduced roughness and surface defects and effective simultaneous protection against wear and corrosion, which can be a cheap and economically and technically feasible substitute for most of the coatings used at the current stage, and to fully utilize its unique advantages to contribute to expanding the application of coated titanium products in various areas under friction and corrosion conditions.

A suitable approach for synergy of high mechanical strength and wear resistance under friction with improved corrosion resistance may be the use of composite ultradisperse electrode materials with nanosized additives, produced by self-propagating high-temperature synthesis SHS, which produce coatings with low roughness and structural defects and a combination of high hardness, nanostructured and amorphous metal phases [21,22,23]. In this aspect, the TiB_2_-TiAl electrodes with nanosized NbC and ZrO_2_ additives produced by authors [23,24,25,26] are particularly promising for the development of protective coatings with improved characteristics and abrasion and corrosion resistance.

In this aspect, TiB_2_-TiAl electrodes with nanosized NbC and ZrO_2_ additives, created by authors [23,24,25,26], are particularly promising for the development of protective coatings with improved characteristics and resistance to abrasion and corrosion. Their use for ESD of steels [23,24,25,26] helps to overcome the main disadvantages of electrospark coatings and obtain dense, uniform layers with low roughness and structural defects, with a combination of high hardness, nanosized and pseudoamorphous metal phases, which creates prerequisites for high tribological and anti-corrosion characteristics. According to authors [27,28], TiB_2_, with its high chemical resistance, hardness (≈34 GPa) and melting point (3000 °C), participates in the improvement of the microstructure of the coatings and prevents the propagation of cracks. Due to its properties, TiB_2_ is widely and effectively used for coatings applied by various methods: thermal, detonation and plasma spraying [29,30,31], 3D laser printing [32], ESD [33,34,35,36]. The authors cited above reported significantly improved characteristics, hardness and wear resistance of coatings containing TiB and TiB_2_. The TiAl bonding component has higher strength and temperature resistance than the titanium base [10,13,14,20] and, as a result of spark discharges, can provide not only strong adhesion to the substrate but also the synthesis of high hardness and wear-resistant aluminum borides and oxides [10,12,13,14,22,37].

The results available in the literature on the use of TiB_2_-TiAl electrode materials show the production of coatings with better characteristics than those obtained using classical hard alloy materials and 2–5 times increased wear resistance of coated steel tools and parts [22,23,25,26] but there is a lack of sufficient data on the use of TiB_2_-TiAl electrodes for coatings on titanium surfaces. Based on the literature, data on the high properties of coatings from TiB_2_-TiAl electrode, as well as our preliminary results [38,39,40] in our previous work [41], the choice of TiB_2_-TiAl electrode was justified and accepted to obtain coatings with improved characteristics and properties and simultaneous protection of titanium surfaces from wear and corrosion. The results obtained in the work [41] confirmed our expectations and, by using contactless local electric spark deposition (LESD) [41,42] with low-energy high-frequency pulses and TiB_2_-TiAl electrodes, continuous dense and uniform coatings with reduced roughness and fewer structural defects, with synthesized new double and triple dispersion-strengthened highly wear-resistant phases, with the presence of pseudoamorphous and nanosized structures and with microhardness up to 13 Gpa were obtained. It was also established that it is possible to predict and control their roughness and thickness by changing the parameters of the LESD mode in the ranges Ra = 1.5 ÷ 3 µm, δ = 6 ÷ 20 µm, respectively. Based on the promising results obtained in [41], in the present work, which appears as a continuation, summary and completion of our previous study [41], the TiB_2_-TiAl electrode was again used.

In this context, the aim of the work is to investigate the influence of ESD process parameters and thickness, roughness and structure on the tribological and corrosion behavior of coatings deposited on titanium Ti-Gr2 with a TiB_2_-TiAl electrode created by SHS, to assess the possibilities for enhancing the corrosion and abrasion resistance of the coated surfaces and to indicate appropriate energy parameters of the ESD process that provide simultaneously increased abrasion and corrosion resistance.

The results of this work suggest filling certain gaps in the scientific field with new data on:

- the use of the ultradisperse TiB_2_-TiAl electrode with nanoscale additives and the contactless local electrospark deposition with low pulse energy to create protective coatings on titanium, for which there is currently no data in the literature;
- the characteristics and properties of the created new coatings with reduced roughness and structural defects, which is a step towards solving the challenge of reducing structural defects and improving the quality of electrospark coatings;
- connections and correlations between the electrical parameters of the regime (resp. pulse energy) → roughness, thickness, microhardness, composition and structure of the coatings → and the tribological and corrosion behavior of the coated titanium surfaces;
- possibilities for synthesis of new nanoscale and amorphous structures and wear-resistant phases from the Ti-Nq Ti-N-C, Ti-B-N, Al-O-N systems on titanium surfaces by selecting appropriate electrical parameters of the LESD mode;
- possibilities for simultaneously increasing the abrasive and corrosion resistance of coated surfaces, which may lead to replacing existing methods for strengthening titanium and its alloys with an easy, ecological, cheap and low-cost method such as ESD and help expand the applications of titanium in many industrial areas where simultaneous protection against wear and corrosion is required.

## 2. Materials and Methods

### 2.1. Electrode Selection

Ultradisperse electrodes were used, created by authors [23,24,25,26] by self-propagating high-temperature synthesis SHS with a composition of TiB_2_-TiAl nano (74%Ti + 12%B + 14%Al), with dispersive reinforced with 7% nano-sized ZrO_2_ and NbC particle additives, which will be referred to as TiB_2_-TiAl for brevity. Electrodes with a diameter of 1.5 mm and a length of 40 mm were obtained by electroerosion cutting from prismatic samples with dimensions of 6 mm × 4 mm × 40 mm.

### 2.2. Substrate

Commercial pure titanium with the trademark Ti-Gr2 (AISI UNS R R56200, VSMPO-AVISMA Corporation, Verkhnyaya Salda, Russia) in the form of square plates with dimensions of 12 mm × 12 mm × 5 mm was used as the substrate. Model plates were obtained from square bars by electroerosion cutting (EDM, Sodick Co., Ltd., Yokohama, Japan) and subsequent grinding to a roughness Ra ≈ 2–2.5 μm.

### 2.3. Research Apparatus

This work is the first study on the use of ESD process and TiB_2_-TiAl electrodes to enhance the abrasion and corrosion resistance of titanium surfaces. The LESD method [41,42] with a rotating electrode, with automatic maintenance of the discharge distance and controlled speed for applying the coatings along the X and Y axes, was used. The schematic diagram of the method and the equipment used are shown in our previous work [41]. The coatings were applied on a mechanized machine type “ELFA”—Sofia, Bulgaria [41,42]—with low pulse energy E = 0.005 to 0.045 J, pulse voltage 100 V, current I = 11.2–24 A, capacitance C = 0.2–5 µF, pulse duration T_i_ = 3–20 µs, pulse frequency f = 5–20 kHz at pulse duty cycle 0.1, deposition speed 0.6 mm/s and three passes of the electrode. The parameters of the deposition regimes that produce uniform, dense coatings with low roughness and surface defects, including amorphous-nanocrystalline structures, were determined based on a preliminary optimization carried out in our previous work [41] and are shown in Table 1.

The parameters of the selected modes in Table 1 are in agreement with the statement by Hassan et al. [36], who use ESD technique with low pulse energy and input power of 40 W for preparation of Ti–TiB–TiB_2_ nanostructured coatings on Ti–6Al–4V substrate, as well as with those in works [23,24,25,26], who use pulses with duration up to 60 μs and low capacity to obtain ultradisperse coatings with amorphous and nanostructured phases.

### 2.4. Research Equipment and Measurement Methodology

#### 2.4.1. Roughness and Thickness of the Coatings

The average roughness of the coatings—Ra, the root means square roughness Rq; the maximum profile height—Rt and the average value of the five highest protrusions and the five deepest depressions of the profile—Rz were measured with a profilometer “AR-132B” (Shenzhen Graigar Technology, Co., Ltd., Shenzhen, China) in two mutually perpendicular directions in five sections in five parallel measurements with a measuring length of 2.5 mm. The arithmetic mean values, standard deviation and confidence interval were determined. Statistically highly different values were rejected using the Grubbs method.

The thickness δ was measured with an indicator with an accuracy of 0.001 mm. The results are the arithmetic mean value of five parallel measurements.

#### 2.4.2. Mechanical Properties of the Coatings

The microhardness (HV) of the coating was measured using a Zwick 4350 hardness tester (Zwick Roell, GmbH & Co., KG, Ulm, Germany) equipped with a diamond prismatic Vickers indenter at a load of 0.2 N for 10 s. Ten parallel measurements were performed. To eliminate the influence of the substrate, measured hardness was calculated according to the method presented in [43].

The coefficients of friction (μ), tangential force and scratch tests of the coatings under progressively increasing normal load from 0 N to 50 N at a rate of 10 N/mm were determined using the “CSM REVETEST Scratch Macrotester” (Anton Paar GmbH—Headquarters, Graz, Austria) with a Rockwell C diamond indenter with a tip radius of 200 μm. The scratch mark was evaluated using light optical Nikon microscope (Tokyo, Japan) with a 14-megapixel digital camera adapted to the microscope and used to capture the image.

The friction tests were performed with a “Finger on disc” tribotester under dry friction with rigidly fixed abrasive particles in planar contact under the following conditions: load 5 N; nominal contact pressure 3.47 N/cm^2^; sliding speed 0.239 m/s; type of abrasive surface—corundum No. 1200.

The following wear characteristics were calculated.

Mass wear—as the difference between the initial mass of the sample “m0” and its mass “mi” after a certain number of friction cycles: m = m0 − mi, mg. The mass of the samples was measured with an electronic balance WPS 180/C/2 (RADWAG Poland, Radom, Poland) with an accuracy of 0.1 mg;

Wear rate: I = m/(P.L), mg/Nm, where m is the wear of the sample for the test time, P—the normal load, L—the friction path travelled;

Wear resistance (reciprocal value of the wear rate).

#### 2.4.3. Corrosion Behaviour of the Coatings

The corrosion tests were performed with a PalmSens4 potentiostat-galvanostat (PalmSens BV, Houten, The Netherlands), PS-Trace 5.9 software and a classic three-electrode cell with a working electrode from the sample and a counter electrode from a Pt plate. All measurements were performed against a reference electrode: Ag/AgCl/3.0 mol/L KCl (E = 0.210 vs. SHE).

A corrosion medium of 3.5 wt.% NaCl was used at a temperature of 20 ± 1 °C, without stirring and deaeration. The samples were degreased with acetone, washed with distilled water and dried with warm air.

Comparative electrochemical measurements were performed, including tests using:

- open circuit potential (OCP), which gives a qualitative idea of the activity of the metal system in a corrosive environment;
- electrochemical impedance spectroscopy (EIS), which provides an opportunity for quantitative characterization of the resistance of the metal system to corrosion when scanning in a frequency range from 10^−2^ to 10^5^ Hz with an AC amplitude of 10 mV;
- potentiodynamic polarization (PDP)/(CVP) when scanning the potential from −0.5 to 2 V vs. Ag/AgCl, to study the active–passive behavior of the coatings.

Each measurement was carried out in three parallel experiments.

The obtained Polarization dependencies, Nyquist plots, and Bode plots were used to estimate the corrosion potential (E_corr_). The corrosion current density (J_corr_), the passivation current density (j_pass_) and the corrosion rate were determined using the Tafel extrapolation method.

#### 2.4.4. Microstructure and Composition of the Layers

The microstructural, morphological analyses and elemental distribution in the coatings before and after the corrosion tests were performed with a metallographic optical microscope “Neophot 22” (Carl-Zeiss, Jena, Germany) and a scanning electron microscope (SEM-EDS) “EVO MA 10 Carl Zeiss” with an integrated EDX energy-dispersive X-ray microanalyzer system “Bruker” (Bruker AXS, Karlsruhe, Germany).

The phase composition was investigated with a Bruker D8 Advance X-ray diffractometer (Bruker AXS, Karlsruhe, Germany) in “CuKά” radiation using the PDF-2 (2009) database of the International Centre for Diffraction Data (ICDD, Newtown Square, PA, USA).

## 3. Results

### 3.1. Coating Characterization–Roughness Ra and Thickness, Structure and Micro-Hardness of Coatings

**Figure 1:**
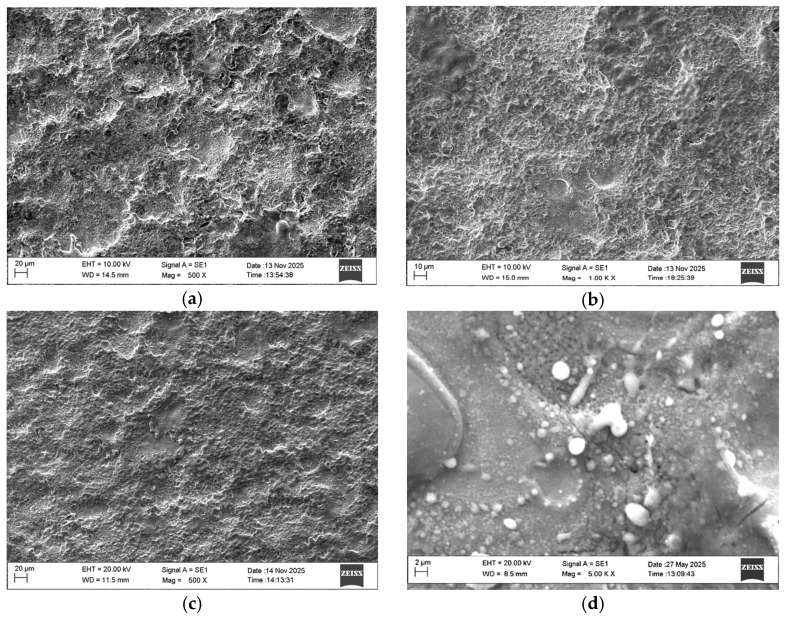
SEM images of the surface of the coatings. (**a**) Sample S1, (**b**) Sample S4, (**c**) Sample S3, (**d**) Sample S5.

The average values of roughness, thickness, minimum and maximum measured microhardness values HV_min_, HV_max_ and the average HV value of the coatings obtained in the present work are shown in Table 2, and Figure 1 shows SEM images of the surface of the coatings deposited in modes 1, 3, 4, 5.

It can be seen that the obtained coatings have high density, acceptable uniformity and repeatability of surface characteristics. Their surface roughness is higher than that of the substrate, but lower than that obtained with traditional methods with vibrating electrodes and higher pulse energy [3,4,5,8]. ESD coatings produced from a WC–8%Co hard metal electrode were tested as a benchmark.

The roughness and thickness of the coatings for the studied energy range vary from Ra 1.6 to 3.36 μm and δ from 9 to 19 μm. The increase in the electrical parameters of the mode (I, C, T_i_) (respectively, the pulse energy) leads to an increase in the roughness parameters and the thickness of the coatings. The coatings obtained at current I = 11.2, 12.8 and 16 A, capacitance C = 0.5 and 2.2 µF and pulse duration T_i_ = 8 and 12 µs—S2, S3 and S4 (Table 2), have a more homogeneous and fine-grained structure, smaller building blocks, higher density and uniformity, and lower roughness than those obtained at the maximum energy used of 0.025 J and capacitance 4.4 µF (Table 1, Figure 1a(S1), and Figure 1d(S5)). In LESD with pulse energy E = 0.015–0.02 J, the depth and size of the initial craters on the titanium surface are lower [6,7,17,34] and, accordingly, the surface roughness, the amount of microcracks and the actual contact area during friction are lower, which creates prerequisites for increasing both the wear resistance during friction and the corrosion resistance of the reinforced surface. Also, prerequisites for improved relief and tribological and anti-corrosion properties are created by the presence of nano-sized particles and the increased amount of amorphous-like and nanostructured phases, registered in [41], as well as the titanium–aluminum alloy, which has a lower melting point than TiB_2_ and “spreads” more evenly on the titanium surface. As can be seen from Figure 1, the main components of the coatings are glass-like zones and individual convex relief formations of transferred incompletely melted electrode material, the amount of which increases with increasing pulse energy above 0.02 J, worsening the uniformity and roughness of the deposited surface. Small, lighter irregular dots and fine needle-like formations are also distinguishable. With properly selected modes, LESD can reduce the initial roughness and improve the topography and quality of titanium surfaces; Table 2(S3).

In the SEM images in Figure 1, darker, relatively smooth areas are observed, which are most likely the metallic matrix of TiAl/TiAl_3_. According to data from many authors [8,9,10,13], Al-containing intermetallics show smoother morphology upon rapid cooling. TiAl_3_ is often formed as an intermetallic matrix or around the boride particles. The small oval lighter irregular dots are most likely borides—TiB and TiB_2_, which have high hardness. The micron sizes of these dots are typical of secondary precipitated TiB/TiB_2_ in Ti and Ti-Al matrix [28,29,30,36]. The fine networks of lighter needle-like formations at the boundaries between the individual areas are likely interparticle phases or oxide phases—Al_2_O_3_, TiO_x_ oxides. The slightly larger convex ridged formations are probably TiB_2_ (primary), TiC_1−x_, TiC_0.3_N_0.7_, or Ti_3.2_B_1.6_N_2.4_. Mixed Ti–B–C–N phases are typical for ultradisperse coatings. In these structures, electroerosion craters and smooth areas of the surface are distinguished. At higher magnification, in the case of the 4.4 µF-deposited films (Figure 1d), the presence of individual microcracks, pores and microscopic irregularities is recorded. Among the particles transferred from the electrode, those with a rounded shape are visible. The presence of the spherical shape of the particles (Figure 1d) indicates that the destruction of the electrode material occurs mainly in the molten state, which is subjected to additional dispersion. The comparison of the obtained coatings shows that the smoothest and flattest surfaces, with the smallest structural elements and the largest number of glass-like regions, have the surfaces modified in modes 2, 3 and 4.

The summary of the obtained results allows us to conclude that the use of pulses with lower energy E ≤ 0.02 J in LESD leads to the formation of a strengthened surface layer with improved roughness, uniformity and reduced surface defects.

**Figure 2:**
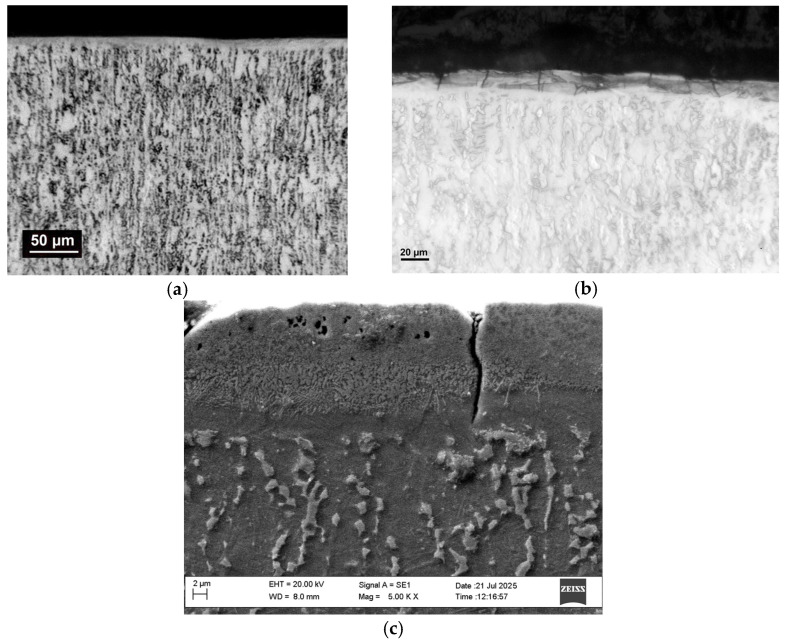
Cross-section microphotographs of microstructure of coating applied by ESD on Ti-Gr2 at sample S3—(**a**), S4—(**b**), and S1—(**c**).

Figure 2 shows an optical image of a cross-section of coating S3 at the lowest pulse energy used.

It can be seen that the resulting coating on sample 3 coating (Figure 2a) has a dense, continuous, compact and uniform microstructure—a white layer, almost free of microcracks, which does not mix with the base. A layer is seen, tightly connected to the base, with a thickness of up to 8–12 µm. In samples S4 and S1—(Figure 2b,c) coatings of greater thickness are observed, but with the presence of cavities and pores.

From the results obtained (Table 2 and Figure 1 and Figure 2), it is established that with increasing pulse energy, the number of microcracks, irregularities and protrusions increase significantly with increasing pulse energy above 0.04 J. And vice versa—reducing energy leads to obtaining uniform coatings with a finer structure and less roughness, but also less thickness. In coatings applied with pulse energy up to 0.02 J, the number of microcracks and irregularities is smaller. As can be seen from Figure 1 and Figure 2, the use of the TiB_2_-TiAl electrode and nano-sized additives at pulse energy up to 0.02 J clearly contributes to the predominant transfer from liquid phase, improving the relief and reducing the structural defects of the coatings. In the coatings deposited with pulse energy above 0.02 J at a higher magnification, a network of microcracks is observed.

The average microhardness values of all coatings are 3–4 times higher than those of the substrate, regardless of the parameters of the LESD regime. The microhardness values of the coatings processed in mode 1-S1 are the highest, followed by those in S4. The microhardness obtained in the individual measurements varies widely, but its average values for the studied coatings are similar ≈11.2–13.5 GPa. Based on the analysis, it can be summarized that the high microhardness of the coatings is due both to the presence of high-hard borides, carbides and nitrides in the composition of the coatings, and to the presence of the ultradisperse, reaching to amorphous-like structure of the matrix of titanium and intermetallic phases. The results obtained by us show that the coatings deposited with TiB_2_-TiAl electrodes with nano-additives significantly increase the microhardness of titanium alloys and confirm the suitability of these coatings for environments with friction and abrasive wear. The results obtained in Table 2 are similar to those of most studies, where microhardness of coatings from different electrode materials in the range of 6–12 GPa is reported, as well as a large scatter of the results of individual measurements. Similar data are presented by Kornienko et al. [15], who obtained the best results at a low pulse energy E = 0.03 J.

### 3.2. Phase Composition

**Figure 3:**
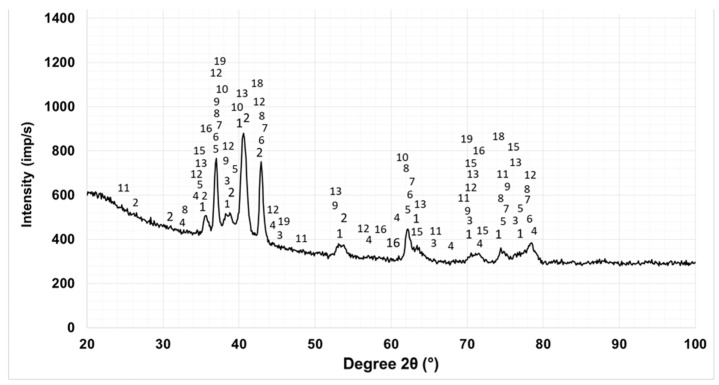
X-ray diffractogram of TiB_2_-TiAl coating sample S5 obtained by LESD.

As the results of XRD analysis presented in our previous work [41] showed, the coatings have a similar phase composition. The main differences are in the intensity and width of the characteristic phase peaks. Figure 3 and Table 3 show the diffractogram and phase composition of sample S5, with a coating deposited at a capacitance C = 4.4 μF. The numbers above each X-ray peak indicate the numbers of the phases from Table 3 that correspond to this peak. The numbers against the phases presented in Table 3 are approximate indicative values of the percentage content of the respective phases and were obtained from the X-ray apparatus used. In Figure 3, at angles 2θ ≈ 35–40°, 53–56°, 61–65°, 70–75°, a noticeable broadening of the characteristic phase peaks is observed. The broadening of the diffraction peaks of the intermetallic phases and Ti reflects the formation of both solid solutions and nanoscale and amorphous-like structures, which is also confirmed by the reduced crystallite sizes of the individual phases in the range of 15–76 nm (Table 4 and Table 5) and is consistent with the results obtained in [38,39,40,41,44,45]. According to those works, as well as to [3,4,5,8,17,45], the extremely high cooling rate of the molten microzones 10^5^–10^6^ °C/s is sufficient to create partial amorphous deposits. The presence of distinct peaks in the extended zones indicates that the rapidly solidified anode–cathode mixture forms both amorphous and fine-grained crystalline structures. As can be seen from Table 5, the crystallite sizes of the individual phases are different for the different regimes used. These differences are probably due to the different individual and complex influence of current, capacitance, duration and frequency of pulses on the crystallite sizes. Obviously, to study these correlations, additional, more extensive studies are needed, which are beyond the scope of the present work.

In all coatings, both the phases of the electrode -TiB_2_, TiAl, and new ones that are not present in the electrode and the substrate were registered. In the composition of the LESD coatings, the presence in small quantities of TiB, Al_2_O_3_, TiN_0.3_, TiCN, Ti_6_O, Ti_2_O, TiC_1−x_, TiC_0.3_N_0.7_, Ti_3.2_B_1.6_ N_2.4_, TiAl, Ti_2_Al, TiAl_3_, traces of AlB_2_, AlN, BN ([41], Table 3), which determine the microstructure and properties of the coatings, was observed. The resulting new phases contribute to improving the bond with the base and to increasing the microhardness and therefore imply higher resistance to corrosion and wear.

Since each of the recorded characteristic peaks at a specific angle 2θ corresponds to several different phases, the table lists all phases recorded in the presence of a minimum of three diffraction peaks.

The obtained surface hardness exceeds the normal value for titanium alloys, but the presence of different phases and structures determines large differences in the individual measured microhardness values (from 7 to 17.7 GPa). The higher hardness of the deposits can be attributed not only to the presence of high-hard phases but also to the ultrafine and amorphous-like structures with fewer defects [41,44]. The highest values are in the upper part of the layer. In depth, the hardness decreases until it equals that of the substrate. The microhardness values of the coatings presented in Table 2 are averaged from 10 measurements. Due to the large differences in the measured values, they cannot serve as an exact comparison of the properties of individual coatings but can be used for an approximate estimate in comparisons. Almost all authors report a significant scatter in the HV values.

### 3.3. Tribological Tests

**Figure 4:**
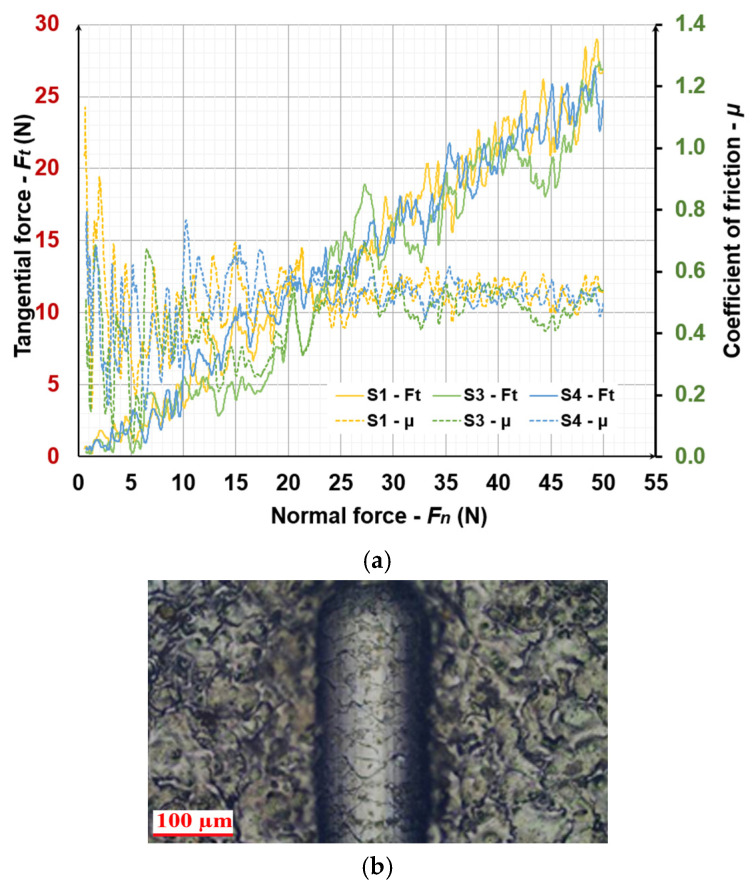
The coefficient of friction (μ), tangential force (Ft), and the scratch track of LESD coatings from TiB_2_-TiAl electrode S1, S3, S4 at progressive load scratching mode with normal force range of 0 N to 50 N at a speed of 10 N/mm. (**a**) The coefficient of friction (μ) and tangential force (Ft); (**b**) A characteristic parts of the track at load 50N of LESD coating of S4.

Figure 4 shows the scratch track, the coefficient of friction (μ) COF, and the tangential force (Ft), of S1, S3, S4 at progressive load scratching mode with normal force range of 0 N to 50 N at a speed of 10 N/mm. Similar data were obtained for the other samples studied.

From the data presented in Figure 4, it can be seen that the values of the friction coefficients μ on the surface of the three samples are very close—about 0.4–0.5 at a load of 50 N. Similarly, the measured values of Ft at maximum load are close and slightly exceed 25 N. The curves of variation of μ and Ft for the three samples have a similar character. The large fluctuations in the signals for S1 are due to the uneven sinking of the indenter with increasing load and the higher roughness of the surface, which is clearly visible in the photographs of the coating (Figure 1a). On the other hand, for S3, the course of variation of μ and Ft with increasing F_N_ is significantly smoother and more uniform. The friction coefficient of the uncoated sample (Ti-Gr2) is slightly higher 0.55–0.6. As can be seen from Figure 4a, with increasing load, the COF shows an increasing trend, increasing to a load of ≈25 N and then remaining almost unchanged up to a load of 50 N. The comparison of the COF values shows that the TiB_2_-TiAl coatings show a relatively lower COF, but the differences are relatively small, no more than 10–15%, despite the significant differences in their roughness parameters.

In sample S1 (Figure 4b), as well as in sample S5, at the very beginning of the load application, cohesive (albeit fine) cracks are observed in the scratch trace. Their size and quantity are larger than those of samples S2, S3 and S4 and increase with increasing load. The reason for this is the lower plasticity of the first two samples, where the coatings were deposited at a capacitance of 4.4 mF. However, all coatings did not show delamination and loss of adhesion at a load of up to 50 N. In samples S2, S3 and S4, no loss of cohesive strength was observed until the end of the test, indicating better plasticity of the coating compared to those in samples S1 and S5. In samples S1, S4 and S5, detachment of individual particles from the coatings with sizes up to several microns was observed, which were probably transferred by unmelted electrode material and were not sufficiently firmly attached to the titanium surface.

**Figure 5:**
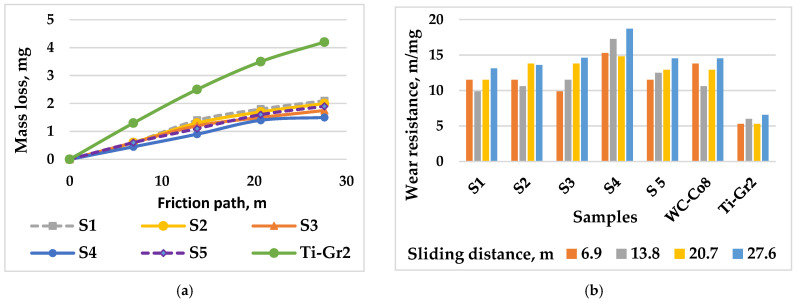
Mass loss and wear resistance of Ti-Gr2 samples with LESD coatings from TiB_2_-TiAl electrode versus sliding distance. (**a**) Mass loss, (**b**) Wear resistance.

Figure 5 shows the effect of pulse energy on the wear and wear resistance of coated samples over time, and Table 6 gives the wear intensity of coated samples. ESD coatings produced from a WC–8%Co hard metal electrode were tested as a wear resistance benchmark.

From Figure 5 and Table 6, it can be seen that regardless of the electrical regime parameters used, the coatings from TiB_2_-TiAl electrode in dry friction show 2–4 times lower wear, respectively higher wear resistance than that of the titanium substrate. This was expected due to the presence of highly wear-resistant TiB, TiN, Ti_3.2_B_1.6_N_2.4_, TiC_0.7_N_0.3_, TiN_0.3_, TiB_2_ and small amounts of AlN, AlB, BN, Al_2_O_3_, Al_2.86_O_3.45_N_0.55_, TiC_0.3_N_0.7_, TiC_1−x_, the nanostructured ZrO_2_ and NbC additives, as well as the fine-grained ultrafine and amorphous structures with fewer defects. The lowest wear is demonstrated by S4, followed by S3 and S2, and the highest S1 and S5, coated with capacitance values C = 4.4 µF. Comparison of the wear of the coated samples shows that the TiB_2_-TiAl electrode coated samples have 1.1 to 1.28 times lower wear than that of the analogous WC-Co8 materials and, accordingly, higher wear resistance. Obviously, the combination of TiB_2_ and TiAl and the nanoscale additives of the electrode allow for a reduction in surface defects and roughness, generation of fine-grained and amorphous structures and two- and three-component highly wear-resistant phases which, according to works [44,45,46], have a major contribution to the obtained higher wear resistance of the coated surfaces compared to that obtained with WC-Co8 electrodes. Changes in the wear rate and wear intensity are similar. Table 7 shows the friction distance of the tested samples under three different wear criteria.

From Table 7, it can be seen that until reaching the wear criterion of 1.5 mg, the longest friction path of 28 m (respectively, the highest durability) is shown by S4, and the coefficient of increase in durability compared to that of the substrate is 3.89.

**Figure 6:**
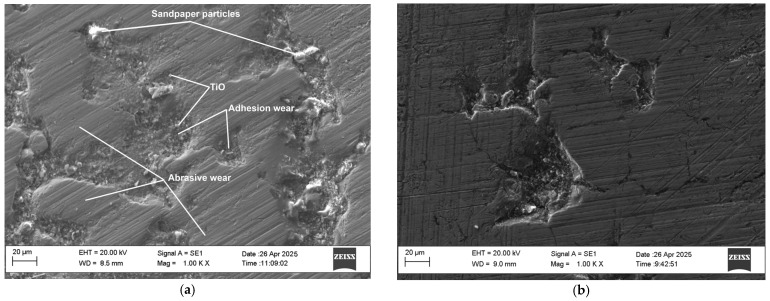
Traces of wear on an uncoated (**a**), and coated S5 (**b**), sample.

The wear process of the uncoated and coated samples was monitored by comparing the wear traces at different friction paths. Figure 6 presents SEM images of the wear traces of an uncoated and coated (sample S5) sample at a friction path of 28 m. From the presented images, it can be seen that the size and depth of the wear craters of the uncoated samples are larger than those of the coated ones. Separate broken-off solid particles from the sandpaper stuck in the surface of the uncoated titanium sample are also observed (Figure 6a), as well as small circles of light particles in the craters, which are probably formed in the friction process and broken-off titanium oxides. Darker small particles separated from the titanium surface by the abrasive action of the sandpaper are also noticeable in the craters. In the coated samples (Figure 6b), the number, size of craters and particles chipped off from the sample surface are significantly smaller, and the scratch marks are shallower. It is clearly seen that the crater formation started from the microcracks in the coating.

Analysis of the friction surfaces showed that the contact surfaces of the friction coated samples are damaged under the action of abrasive, adhesive and oxidative wear mechanisms. The development and growth of craters from adhesive wear, as well as abrasive wear and chipping of particles from the coated samples starts from the irregularities and microcracks. In the uncoated samples, adhesive wear is predominant and at a friction distance between 7 and 14 m, relatively deep craters and scratches are already present. In the coated samples, chipping of particles from the transferred unmelted electrode material is observed at the beginning of friction, as well as those from the highest peaks of the microirregularities. The separated particles are driven between the friction surfaces and contribute to the acceleration of wear until the surface is smoothed. The appearance of formed deeper adhesion craters begins after the 20th meter.

Therefore, LESD does not prevent wear, but only slows down and reduces its development over time. The higher wear rate of the coated samples at the beginning of the friction process—to the 7th and 14th meter (Table 6)—is mainly due to the tensile stresses in the surface layer, which are characteristic of all electrospark coatings and to the detachment of the particles carried by brittle fracture of the electrode, which act as an abrasive. After the removal of the uppermost uneven layer of the coating and the formed abrasive particles from the friction zone, a period of steady state occurs, in which, as a result of the high microhardness of the boride, nitride and carbide phases and the strength of the Ti-Al metal matrix, wear intensity slightly decreases.

The obtained results show that the influence of the electrode material is also related to the LESD mode. The classical surface microgeometric indicators (R_a_, R_z_, R_max_) in this case do not reflect the actual situation of the frictional contact. The coatings of S4 with higher values of R_a_, R_z_, R_max_ have higher wear resistance than those of S2 and S3 with low roughness. The higher roughness of the coatings obtained in mode 4, Table 2, suggests a decrease in wear resistance, but this is apparently compensated by the greater thickness and concentration of the wear-resistant phases in the layer, as well as by the higher degree of dispersion and non-equilibrium, i.e., the increase in the pulse energy has led to an increase in the wear resistance of the coatings. With a further increase in the pulse energy—S1 and S5—regardless of the greater layer thickness and the higher amount of wear-resistant phases, the wear resistance shows a tendency to decrease. From the obtained results, it can be concluded that increasing the pulse energy to 0.02 J and the values of the electrical parameters to I = 16 A, C = 2.2 µF, T_i_ = 12 µs increases the durability of the coated surface. However, with a further increase in energy, due to the higher roughness and irregularities and structural defects of the coatings, the relative wear resistance gradually begins to decrease. The described wear mechanism, as well as the obtained data on the wear resistance of the coatings are close to those obtained by Koga et al. for plasma sprayed amorphous coatings containing TiB_2_ [47]. These results show that we have obtained a promising coating with higher performance properties than surfaces coated with classical WC-Co hard alloys. Coatings obtained with lower pulse energies (S2, S3 and S4) reduce wear intensity compared to uncoated alloys. In this context, they can be used both to reduce surface defects and to increase the durability of titanium friction surfaces and parts exposed to abrasive wear.

### 3.4. Corrosion Tests

The behavior of TiB_2_-TiAl electrode layers in contact with 3.5% NaCl corrosion medium without external polarization was monitored by measuring the open circuit potential (OCP). All samples demonstrated an open circuit potential (OCP) about 150–200 mV more positive than that of the Ti-Gr2 substrate (Table 8). The OCP values of the samples coated under different regimes are located in a relatively narrow potential range of about 120 mV. They are about 200 mV more positive for all coatings than for the uncoated Ti-Gr2 substrate. From the table, it is seen that the thickness of the substrate corrosion is 1–2 orders of magnitude lower than that of sample S3, and the wear rate of the coated samples is an order of magnitude lower than the uncoated ones. These results indicate reduced surface activity after the application of the layers.

Electrochemical impedance spectroscopy provides a quantitative characterization of the corrosion resistance of a metal system. The tests were performed at an OCP amplitude of 10 mV with samples with TiB_2_ layers and an uncoated Ti-Gr2 substrate. The results are presented in Figure 6 as Nyquist and Bode plots.

**Figure 7:**
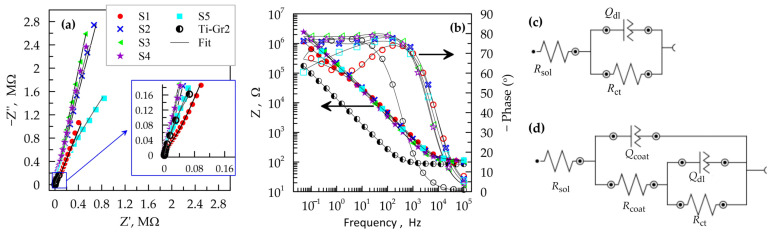
EIS experimental results (symbols) and equivalent circuits for fits (black line): (**a**) Nyquist plot; (**b**) Bode plots of impedance module (filled symbols) and phase angles (unfilled symbols); (**c**) EIS equivalent circuits to describe the behavior of the Ti-Gr2 substrate; (**d**) EIS equivalent circuits to describe the behavior of the TiB_2_–TiAl electrode layers. The legend presented in (**a**) is also valid for (**b**).

The Nyquist plot of the Ti substrate is in the form of an incomplete arc (Figure 7a—inset), which is characteristic of metals in a passive state. The increased arc radius indicates improvement of the corrosion resistance and dominance of the capacitive behavior of the coatings. The Nyquist plots (Figure 7a) for S2, S3 and S4 are similar to the strongly pronounced capacitive behavior of protective passive layers. The Bode plots show that in the frequency range 0.01–100 Hz, the impedance of the coated samples is about one order of magnitude higher than that of the substrate, which gives an additional indication of the effectiveness of the LESD coatings from the TiB_2_-TiAl electrode. Therefore, the formed coatings show significantly better barrier ability than the uncoated titanium substrate.

The Bode plot of the titanium substrate (Figure 7b) shows typical behavior of a passive metal surface with a single time constant, which is described by the simplest Randall electrochemical circuit, consisting of solution resistance R_S_ in series with a block of electric double layer capacitance Q_dl_ and a charge transfer resistance R_ct_ (Figure 7c). This equivalent circuit has been frequently used to describe the corrosion behavior of titanium and its alloys [13,15,27,48]. In contrast to the substrate, all LESD layers demonstrate a circuit with two-time constants (two maxima in the frequency dependence of the phase shift of the Bode plot). The equivalent circuit in this case is presented in Figure 7d. It has been used in the characterization of titanium alloys and other passive metal systems with highly porous and highly protective passive layers [49,50]. In this case, the circuit is supplemented with the capacitance (Q_coat_) and the resistance (R_coat_) of the coating. All capacitances are represented as a constant phase element Q, instead of pure capacitance, since the phase angle does not exceed −80° in the entire frequency range studied (Figure 7). Figure 7 shows good agreement between the experimental values (represented by symbols) and the fits (black lines) of the used equivalent circuits (Figure 7c,d). The calculated values of the components in the equivalent circuits are presented in Table 9.

In general, higher values of R_ct_ are associated with the difficulties of electrochemical reactions (anodic dissolution). All coated samples have one to four orders of magnitude higher R_ct_, which indicates that TiB_2_-TiAl coatings can effectively protect the titanium substrate. The highest values of both R_ct_ and R_coat_ are found in S3, whose layer was obtained at the lowest pulse energy, capacitance and pulse duration used in this study. The R_ct_ values for S2 and S4 are close to those of S3 and exceed 10^10^ Ω. These layers were also deposited at low to medium pulse energy and capacitance like S3 (Table 1). Samples S1 and S5 deposited at the highest capacitance of 4.4 µF as well as high to medium pulse energy, have two and three orders of magnitude lower R_ct_, respectively. However, R_ct_ of the titanium substrate is the lowest, with the value remaining below 1 MΩ.

The resistance R_coat_ of samples with TiB_2_-TiAl coating increases the total resistance by an additional over 100 kΩ. These results clearly demonstrate the high corrosion resistance of the deposited layers, of which the highest is that obtained in mode 3. The coating obtained by this mode is smooth, uniform and continuous, almost without structural defects and with the most amorphous and nanoscale phases, as well as with the lowest microhardness of 11.2 GPa and thickness of 9 µm. The lowest R_coat_ have S1 and S5, which are characterized by the highest thickness and microhardness, with the highest content of wear- and corrosion-resistant borides, nitrides and intermetallic phases, but also with the highest roughness, unevenness and structural defects.

**Figure 8:**
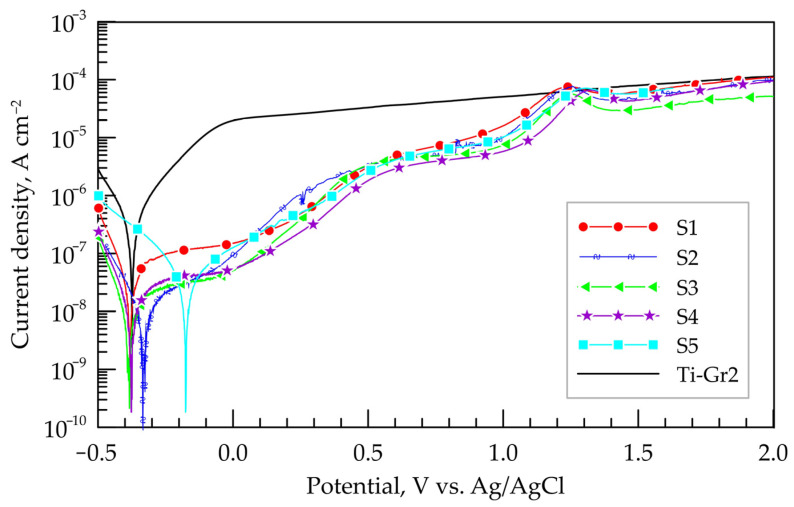
Polarization dependences of TiB_2_-TiAl coatings on Ti-Gr2 in 3.5% NaCl.

Figure 8 presents polarization dependences of Ti-Gr2 samples coated with TiB_2_-TiAl electrodes in 3.5% NaCl. The main corrosion parameters obtained from them are the corrosion potential (E_corr_), the corrosion current density (J_corr_), the polarization resistance (R_p_) and corrosion rate (CR), and their values are presented in Table 7. These parameters are criteria for the protective properties of coatings. Thus, a more positive E_corr_ usually indicates a tendency towards a passive state as well as higher values of R_p_, and lower values of J_corr_ and CR of the coated samples indicate better corrosion resistance.

Unlike the open circuit potentials, the corrosion potentials of the samples approach that of the titanium substrate Ti-Gr2, with only that of the layer of mode 5 remaining more positive. Most likely, this discrepancy is due to the short cathodic polarization at −0.5 V vs. Ag/AgCl, performed before starting the polarization test. The corrosion current density J_corr_ of all coated samples is one or two orders of magnitude lower than that of the titanium substrate (Table 7). The lowest values of J_corr_ and CR are again recorded for S3—two orders of magnitude lower than that of the substrate, followed by S2 and S4. The highest current density is in S1 and S5. These results are consistent with those from the EIS analysis. As presented above (Table 2), the coatings of S3 have lower thickness and roughness parameters, lower microhardness than that of S1, S4 and S5 and smaller amounts of high-hard wear-resistant phases and structural defects but more amorphous and nanoscale phases than those in the coatings deposited with higher pulse energy (capacity C = 4.4 µF) S1 and S5, in which the thickness of the coatings and roughness are the highest, and the structural defects such as cracks, micropores and cavities are the most numerous (Figure 2). Apparently, the presence of microcracks suggests access to the surface of the titanium substrate, and the higher roughness and irregularities suggest a larger contact area, which facilitates the flow of current and, accordingly, corrosion.

During anodic polarization in 3.5% NaCl, the layers undergo complex multi-stage transformation processes in the passive oxide layers. At potentials above 0.5 V vs. Ag/AgCl, the dependences of the layers are superimposed, and at potentials above 1.2 V vs. Ag/AgCl they are aligned with that of the substrate. This behavior indicates a similar composition of the surface layers formed under the different regimes. Neither the TiB_2_-TiAl layers nor the Ti-Gr2 substrate show a tendency to local corrosion, which can be expected for passive metals in the presence of chlorides and high anodic polarization.

**Figure 9:**
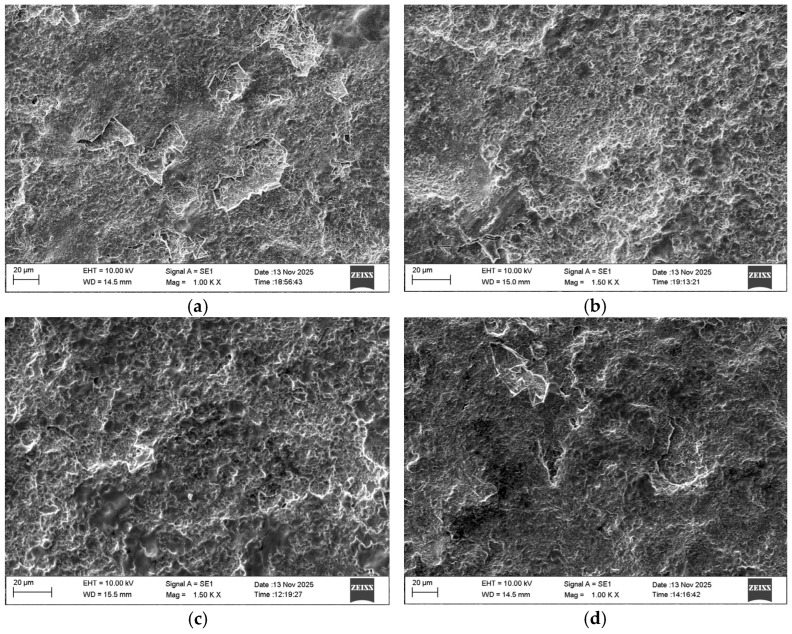
SEM images of the LESD coating surfaces after corrosion tests. (**a**) Sample S1, (**b**) Sample S2, (**c**) Sample S4, (**d**) Sample S5.

Figure 9 presents typical SEM images of coated surfaces after corrosion tests. SEM observations showed that after the corrosion tests on the S3 surface with coatings at the lowest pulse energy used, no particular changes were observed in the surface and relief of the coatings. After the corrosion tests on the surface of S2 and S4 (Figure 9b,c) the appearance of white dots, threads and needle-like formations is observed. In the secondary electron mode (SE), this is an indication of the formation of phases with lower electronic conductivity, such as aluminum oxides. In addition to similar formations, individual shallow depressions with light borders are also observed on samples S1 and S5 (Figure 9a,d). These local destructions appear to be the result of the operation of a microcorrosion galvanic cell, in which energy-rich zones are preferentially dissolved.

In summary, the results obtained from the corrosion tests show that the protective properties of the coatings of samples S2, S3 and S4 are similar and significantly better than those of samples S1 and S5. The obtained ultradisperse uniform coatings with amorphous-nanocrystalline structures and high-hard and chemically resistant phases such as titanium nitrides, borides and carbonitrides, and ternary dispersion-strengthened ones such as Ti_3.2_B_1.6_N_2.4_, Al_2.86_O_3.45_N_0.55_ serve as a barrier to the metal with respect to corrosion agents, providing high protection of the titanium substrate. The presence of corrosion-resistant nanosized particles ZrO_2_ and NbC also contributes to improving the uniformity of the coating and increasing corrosion resistance. In addition, the small quantities Ti_2_O, Ti_6_O and Al_2_O_3_ synthesized in the LESD process and incorporated in the composition of the coatings also contribute to effective protection against corrosion in the aggressive chloride environment. The layers of aluminum oxide on them are impermeable to oxygen and thus protect the titanium.

The obtained results allow us to assume that from the point of view of corrosion resistance, low roughness and uniformity, the presence of amorphous-nanocrystalline structures and the absence of structural defects are more significant factors than thickness, microhardness and the presence of larger amounts of high-hard boride and nitride phases. Obviously, porosity and structural defects are closely related to both the wear resistance and the corrosion properties of LESD coatings. Of the pulse parameters, the strongest influence is the capacitance, followed by that of t_i_ on the current I.

The coatings obtained at capacitance values of 4.4 µF have cracks and pores (Figure 2b,c) that deteriorate corrosion resistance despite their greater thickness and higher content of high-hard and corrosion-resistant phases. However, despite these defects, local corrosion does not develop even at an anodic polarization up to 2 V. Therefore, it can be assumed that the highest corrosion resistance achieved in these studies is primarily a result of the better uniformity and the smaller amount of pores and microcracks in the coatings deposited in the modes with average pulse energy 0.013 J and 0.02 J. Achieving defect-free coatings by LESD methods is a challenge due to their inherent porosity and microcracks. The present work offers a possible solution to this challenge by using the TiB_2_-TiAl electrode and the LESD process in modes with capacitance values up to 2 µF and pulse durations of 8 and 12 µs, making it possible to obtain coatings almost free of structural defects and with many times increased corrosion, abrasion and adhesion resistance.

The coatings of the TiB_2_-TiAl–nano electrode material created by self-propagating high-temperature synthesis show up to four times higher wear resistance and up to three orders of magnitude higher corrosion resistance than that of the Ti-Gr2 substrate. These results demonstrate that we have obtained promising corrosion resistant coatings, providing corrosion protection of the material and significantly higher performance properties than those of coatings from classical WC-Co hard alloys. The TiB_2_-TiAl coatings can be used to increase the durability of titanium surfaces subjected to corrosive and abrasive wear.

## 4. Discussion

The summary of the obtained data allows us to conclude that the influence of the electrical parameters of the mode on the characteristics and properties of the coated and uncoated surfaces is complex and contradictory. It is established that with an increase in the values of the electrical parameters of the mode for LESD that determine the energy of individual pulses, (current I, capacitance S, pulse duration Ti,) the thickness of the coatings monotonically increases. The microhardness also shows a tendency to slightly increase. The number of newly formed wear-resistant phases and nanoscale and pseudoamorphous structures synthesized in the spark discharges process also increases. As shown in our previous work [41], the influence of capacitance C is the strongest, followed by the pulse duration Ti. On the other hand, the transfer of incompletely melted electrode material increases in parallel, as well as the roughness, unevenness, and the number of structural defects (cavities, micropores, and cracks), which is unfavorable in terms of the tribological and corrosion characteristics of the coatings. At LESD with the maximum energy used of 0.025 J and a capacity of 4.4 µF, the roughness of the coatings increases to Ra ≥ 3 µm, their thickness reaches almost 20 µm, but their unevenness and the number of structural defects significantly increase. To obtain a coating with lower roughness and structural defects, a lower pulse energy is required, but in this case the thickness of the coatings and the amount of high-hard phases and nanoscale and pseudoamorphous structures will also be lower. It was found that up to values of the electrical parameters I = 11.2–16 A, capacitance C = 0.5–2.2 µF, pulse duration Ti = 8–12 µs and pulse energy to 0.02 J, the coatings have the most favorable characteristics, composition and structure. This finding was confirmed by the results of the tribological tests, in which it was found that with an increase in the capacitance to 2.2 µF and the pulse energy to 0.02 J, the wear resistance of the coated samples also increases until reaching a maximum, after which at a capacity of 4.4 µF a tendency to its decrease begins. Obviously with an increase in the energy to 0.02 J, the negative influence of the roughness and structural defects of the coatings is overcome by increases in the number of highly hard and wear-resistant, nano-sized and pseudo-amorphous phases in the coated surfaces. At values of capacitance C ≥ 4.4 μF, however, the influence of increased roughness and surface defects begins to prevail and wear resistance shows a tendency to decrease. In corrosion tests, however, the highest corrosion resistance was shown by samples with coatings at a lower pulse energy—0.013 J, with smooth, uniform and continuous coatings almost without structural defects. The fine-grained structure and the absence of structural defects have a clear predominant influence on corrosion resistance, and are more significant factors than thickness, microhardness and the presence of larger amounts of high-hard boride and nitride phases, although the presence of highly wear-resistant compounds in the coatings also increases corrosion resistance. However, it is obvious that porosity and structural defects are closely related to both the wear resistance and the corrosion properties of LESD coatings. From the point of view of achieving simultaneous high and maximum possible corrosion and wear resistance for the conditions under consideration, sample 4 emerges as the most suitable—with a coating at a current of 16 A, at a capacity of 2.2 µF, a pulse duration of 12 μs and an energy of 0.02 J.

In conclusion, it can be stated that the results obtained in this study show that the use of the TiB_2_-TiAl electrode and the LESD method can lead to a significant improvement in the protective properties of coatings against simultaneously wear and corrosion. The obtained ultradisperse coatings with 3–4 times higher hardness, with the presence of pseudoamorphous and nanoscale structures and newly synthesized high-hardness and chemically resistant phases such as titanium nitrides and borides and ternary dispersion strengthened such as TiCN, Ti_3.2_B_1.6_N_2.4_, Al_2.86_O_3.45_N_0.55_ serve as a barrier reducing wear and corrosion of the titanium substrate. Despite the positive results, it was found that the coatings are still not homogeneous enough and there are no completely uniform data on their microhardness. Heterogeneity is typical for multicomponent coatings [1,3,5,6,32,33,34] but it does not allow for the establishment of a clear dependence of the microhardness on the parameters and energy of the pulse. The significant differences in the measured values of the universal nanohardness HU and the microhardness HV within the same coating, obtained both in the work [41] and in this work, are obviously due not only to the differences in the hardness of the individual components, of the changed initial chemical composition, of the oxidation, dissociation of TiB_2_ and the formation of new compounds, but also to the uneven distribution of individual phases and elements and heterogeneity in the coating surface.

The obtained coatings can be made even more resistant to corrosion and wear by further reducing the roughness, unevenness and number of microcracks, increasing density and homogeneity and increasing the number of nanoscale structures and synthesis of new phases by searching for different approaches. There are ample opportunities for further research aimed at improving the characteristics of electrospark coatings through additional hard coatings of other compounds and alloys, additional processing with other methods (laser beam [14], surface plastic deformation), etc. For example, conducting further research using additional processing by subsequent polishing by low-temperature or ultrasonic surface plastic deformation, which have proven their capabilities for improving the quality and structure of the surface [51,52], could lead to even higher results.

## 5. Conclusions

The coatings obtained by LESD on titanium (Gr2), with a TiB_2_-TiAl electrode created by self-propagating high-temperature synthesis, dispersedly strengthened with nanosized additives of NbC and ZrO_2_, are uniform with low roughness, increased to 13 GPa microhardness, and minimized surface defects with synthesized new wear-resistant and intermetallic compounds such as TiB, Ti_3.2_B_1.6_N_2.4_, TiC_0.7_N_0.3_, TiN_0.3_, TiB_2_, AlN, AlB, BN, Al_2_O_3_, Al_2.86_O_3.45_N_0.55_, TiC_0.3_N_0.7_, TiC_1−x_, TiAl_3_ and ultrafine and amorphous-crystal structures.

With a pulse energy of up to 0.02 J, a relatively low capacitance of 0.5–2.2 µF and short pulses of 8–12 µs, a crack-free coating with high adhesion can be obtained, with roughness and thickness that can be changed by changing the pulse energy in the range of roughness Ra 1.6–2.5 µm, thickness 9–16 µm and microhardness 12–13 GPa. Coatings applied at lower pulse energy have lower roughness and structural defects, improve surface quality and reduce the friction coefficient of the coated surfaces by up to 10–20%. They increase the frictional wear resistance by three to four times, reduce the corrosion rate and increase the corrosion resistance of the modified surfaces by one to two orders of magnitude compared to those on a titanium base.

The main factors that provide an increase in the anti-corrosion properties of coatings are their density and roughness, as well as ultradisperse and metastable amorphous-crystalline phases, the amount and distribution of which determine increased corrosion resistance. Studies have shown that corrosion occurs from defects such as pores and microcracks. Therefore, conducting future research aimed at improving the uniformity and density of coatings and reducing pores and microcracks is crucial and can significantly improve the corrosion resistance of the strengthened surface.

The study showed that LESD with appropriate electrodes and modes has significant potential for improving corrosion and abrasion resistance and expanding the application of titanium surfaces. The results obtained are the basis for subsequent research aimed at transforming ESD into a powerful tool for creating coatings with improved surface properties. Conducting experiments in this direction may allow for the control and optimization of the process for creating coatings with predictable composition, structure and properties, as well as determining ways to control the composition, structure and mechanical and operational properties of the coated surfaces.

## Figures and Tables

**Table 1:** Parameters of the selected modes for LESD.

Designation of Samples	Current, I, A	Capacitance C, μF	Pulse Duration T_i_, μs	Frequency f, kHz	Pulse Energy E, J
S1	16.0	4.4	12	8.33	0.025
S2	11.2	0.5	12	8.33	0.013
S3	12.8	0.5	8	12.5	0.013
S4	16.0	2.0	12	8.33	0.020
S5	12.8	4.4	8	12.5	0.020

**Table 2:** Roughness parameters, thickness δ, minimum HV_min_, maximum HV_max_ and average microhardness HV of LESD coatings from TiB_2_-TiAl electrode.

Sample	Ra, µm	Rz, µm	Rt, µm	δ, µm	HV_min_	HV_max_	HV, GPa
S1	3.36	10.94	15.93	19	7.93	16.87	13.5
S2	2.07	7.53	9.05	11	7.45	14.25	11.9
S3	1.63	6.39	8.65	9	7.12	13.93	11.2
S4	2.45	9.05	11.66	16	7.64	17.78	13.2
S5	2.57	9.89	13.86	13	7.88	15.16	12.3
WC-Co8	2.68	10.14	11.33	12	7.65	14.54	11.8
Ti-Gr2	2.07	5.62	5.67		2.35	3.59	3.4

**Table 3:** Phase composition of the coatings S5.

N	Main Phases	N	Phases in Small Amounts	N	Traces of Phases
**1**	Ti ≈ 32.9%	**7**	TiN ≈ 4.4%	**14**	Al ≈ 0.8%
**2**	AlTi_3_ ≈ 2.6%	**8**	Ti_3.2_B_1.6_N_2.4_ ≈ 15%	**15**	TiC_0.7_N_0.3_ ≈ 0.8%
**3**	AlTi ≈ 1.8%	**9**	Ti_6_O ≈ 3.3%	**16**	AlN ≈ 2.1%
**4**	TiB_2_ ≈ 4.5%	**10**	Ti_3_O ≈ 1.7%	**17**	AlB ≈ 0.9%
**5**	TiN_0.3_ ≈ 5.3%	**11**	Al_3_Ti ≈ 2.6%	**18**	BN ≈ 1.1%
**6**	TiB ≈ 8.8%,	**12**	Al_2_O_3_ ≈ 2.5%	**19**	Al_2.86_O_3.45_N_0.55_ ≈ 2.4%
		**13**	Ti_2_O ≈ 3%,	**20**	TiC_0.3_N_0.7_ ≈ 3.5%

**Table 4:** Crystallite size, of characteristic phases of LESD coatings from TiB_2_-TiAl electrode—S5.

Phases	Ti	TiN_0.3_	TiN	TiC_1−x_	TiC_x_N_y_	TiB	TiB_2_	TiAl_3_	Ti_2_O	Al_2_O_3_	Ti_3.2_B_1.6_N_2.4_	TiAl	Ti_2_Al
**Size, nm**	39	36	29	26	24	35	68	41	39	51	42	42	32

**Table 5:** Dimensions of the crystal lattices (Å), and crystallites size—CS (nm) of characteristic phases of LESD coatings from the TiB_2_-TiAl electrode.

Phases	α-Ti,	TiN_0.3_	AlTi_3_	TiB_2_	Ti_3.2_B_1.6_N_2.4_	Ti_2_O	Al_2_O_3_
Angle 2θ	35.7; 38.8; 40.7; 53.5; 63; 7; 75.4; 76.8; 78.6	35; 37.5; 39.5; 52.2; 62.5; 69.2 75.5; 77	26.33; 31.1; 35.65; 38.9; 39.4; 40.8; 43.05; 53.8	27.7; 33.38; 34.2; 44.6; 56.9; 61.1; 68.5; 72; 78.6	33.36; 37; 42.95; 62.25; 74.5; 78.5	33.6; 35.65; 38.45; 40.7; 53.2; 63.75; 70.5; 76.6; 77.15; 78.5	19.5; 32.1; 35.65; 37.6; 39.2; 43.05; 44.5; 45.6; 50; 56.7; 60.5; 66.8; 71.4; 75.3
Sample S1,							
Lattice, Å	a = 2.936,	a = 2.969,	a = 5.768,	a = 3.027,	a = 4.23	a = 2.9194,	a = 7.947
	c = 4.652	c = 4.783	c = 4.6424	c = 3.211		c = 4.7130	
CS, nm	32	45	46	21	42	44	76
Sample S2,							
Lattice, Å	a = 2.949,	a = 2.956,	a = 5.764,	a = 3.032,	a = 4.2350	a = 2.9194,	a = 7.9320
	c = 4.684 27	c = 4.77	c = 4.664	c = 3.219		c = 4.7130	
CS, nm	22	36	36	45	45	46	54
Sample S3,							
Lattice, Å	a = 2.956,	a = 2.968,		a = 2.96,	a = 4.2430		a = 7.956
	c = 4.692	c = 4.78		c = 3.31			
CS, nm	26	43		15	-		46
Sample S4,							
Lattice, Å	a = 2.945,	a = 2.963,	a = 5.768,	a = 2.96,	a = 4.220	a = 2.9194,	a = 7.955
	c = 4.683	c = 4.78	c = 4.642	c = 3.32		c = 4.7130	
CS, nm	27	31	33	21	38	39	52

**Table 6:** Wear intensity of Ti-Gr2 LESD with TiB_2_-TiAl electrode.

	**Sliding distance, m**			
	6.9	13.8	20.7	27.6
**Samples**	**Wear intensity, mg/m·10^−2^**			
S1	8.7	10	8.6	7.5
S2	8.71	9.3	8	6.1
S3	8.6	8.5	7.1	6.3
S4	6.5	6.4	6.7	5.2
S5	6.8	8.2	7.8	7.2
Ti-Gr2	18.8	17.8	16.9	15.6
WC-Co8	7.2	9.4	7.7	6.9

**Table 7:** Durability of LESD samples at different criteria of wear.

Friction Path, m/ The Durability Enhancement Factor; Wear, mg	Uncoated Ti-Gr2	LESD with TiB2-TiAl					LESD with WC-Co8
		S1	S2	S3	S4	S5	
1.5	7.2/1	17/2.36	17.6/2.43	21/2.92	28/3.89	18.2/2.57	18.1/2.51
1	5.5/1	11/2	11.5/2.1	12/2.2	19/3.45	13/2.36	12/2.2
0.5	2.2/1	5/2.3	5.2/2.4	5.5/2.5	10/4.54	5.9/2.7	5.85/2.66

**Table 8:** Corrosion parameters of LESD coatings from TiB_2_-TiAl electrode, obtained from polarization dependences taken in 3.5% NaCl.

Samples	OCP (600 s), V	E_corr_, V	J_corr_, A/cm^2^	j_pass_, A/cm^2^	R_p_, Ohm	CR, mm/year
S1	−0.076	−0.382	1.11 × 10^−7^	-	8.58 × 10^7^	12.9 × 10^−5^
S2	−0.194	−0.336	1.35 × 10^−8^	4.76 × 10^−6^	3.08 × 10^7^	1.57 × 10^−5^
S3	−0.143	−0.388	9.25 × 10^−9^	5.02 × 10^−6^	2.83 × 10^7^	1.08 × 10^−5^
S4	−0.158	−0.378	1.68 × 10^−8^	4.15 × 10^−6^	2.06 × 10^7^	1.95 × 10^−5^
S5	−0.147	−0.170	7.84 × 10^−8^	4.24 × 10^−6^	3.45 × 10^6^	9.11 × 10^−5^
Ti-Gr2	−0.380	−0.375	3.97 × 10^−7^	-	8.50 × 10^5^	4.62 × 10^−4^

**Table 9:** Equivalent electrical circuit parameters for TiB_2_–TiAl coatings at OCP.

Samples	R_sol_, Ω	R_ct_, Ω	Q_dl_, µS s^n^	n_dl_	R_coat_, kΩ	Q_coat_, µS s^n^	n_coat_
S1	104	73.2 × 10^7^	1.400	0.714	85.3	0.791	0.864
S2	94.8	63.0 × 10^9^	0.252	0.740	249	0.697	0.890
S3	102	93.0 × 10^9^	0.177	0.740	384	0.877	0.907
S4	113	43.0 × 10^9^	0.221	0.705	273	0.894	0.899
S5	114	20.9 × 10^6^	0.543	0.560	153	0.837	0.876
Ti-Gr2	84.3	8.92 × 10^5^	14.70	0.872	–	–	–

## Data Availability

The original contributions presented in this study are included in the article. Further inquiries can be directed to the corresponding authors.
